# Transperineal MRI-US Fusion-Guided Biopsy with Systematic Sampling for Prostate Cancer: Diagnostic Accuracy and Clinical Implications Across PI-RADS

**DOI:** 10.3390/cancers17172735

**Published:** 2025-08-22

**Authors:** Valèria Richart, Meritxell Costa, María Muní, Ignacio Asiain, Rafael Salvador, Josep Puig, Leonardo Rodriguez-Carunchio, Belinda Salinas, Marc Comas-Cufí, Carlos Nicolau

**Affiliations:** 1 Radiology Department, Hospital Clinic, 08036 Barcelona, Spain; 2 Urology Department, Hospital Clinic, 08036 Barcelona, Spain; 3 Radiology Department, Hospital Clinic and IDIBAPS, 08036 Barcelona, Spain; 4 Department of Pathology, Hospital Clinic, 08036 Barcelona, Spain; 5 Department of Computer Science, Applied Mathematics and Statistics, University of Girona, 17004 Girona, Spain; 6 Radiology Department, Hospital Clinic, IDIBAPS, University of Barcelona (UB), 08036 Barcelona, Spain

**Keywords:** MRI-US fusion, prostate cancer, PIRADS, transperineal biopsy, ISUP, targeted biopsy

## Abstract

**Background/Objectives**: Magnetic resonance imaging (MRI) and MRI–ultrasound (US) fusion-targeted biopsy have improved prostate cancer diagnosis, particularly for clinically significant disease. However, the added value of combining systematic biopsy with targeted biopsy remains debated. This study aimed to evaluate the diagnostic accuracy of MRI–US fusion-targeted and systematic transperineal biopsies in detecting prostate cancer and explore the correlation between PI-RADS score and histology. **Methods**: We retrospectively analyzed 356 patients with 452 MRI-detected lesions who underwent both MRI–US fusion-targeted and transperineal systematic biopsies between 2020 and 2023. Clinically significant prostate cancer (csPCa) was defined as International Society of Urological Pathology (ISUP) grade ≥ 2. Diagnostic performance metrics (sensitivity, specificity, and accuracy) were calculated for each technique using the combined result as a reference. Subgroup analysis was performed for patients under active surveillance. **Results**: Prostate cancer was diagnosed in 323 of 452 lesions (71%) and csPCa in 223 lesions (49%). Targeted biopsy demonstrated higher sensitivity (93.7%) and accuracy (79.9%) than systematic biopsy (85.7% sensitivity and 77.6% accuracy), although systematic biopsy provided slightly higher specificity. Systematic biopsy alone identified 8.2% of PCa cases missed by targeted biopsy and upgraded 9.9% of lesions to csPCa. csPCa detection increased with PI-RADS score (23% in PI-RADS 3 and 73% in PI-RADS 5). In active surveillance patients, csPCa was found in 65% of lesions. **Conclusions**: MRI–US fusion-targeted biopsy improves csPCa detection, but systematic biopsy remains valuable, especially for identifying additional or higher-grade disease. The combined approach provides an optimal diagnostic yield, supporting its continued use in both initial and repeat biopsy settings.

## Simple Summary

Early and accurate detection of aggressive prostate cancer is essential to guide treatment and avoid unnecessary procedures. Magnetic resonance imaging (MRI) has become a valuable tool to help identify suspicious areas in the prostate, and targeted biopsy methods guided by ultrasound (US) allow for sampling of these specific regions. In this study, we investigated whether combining MRI-US fusion-targeted biopsy with a systematic approach improves cancer detection. We found that MRI-US fusion-targeted biopsy was more effective in finding clinically important cancers, but the systematic method still found some cases that the targeted method missed. This combined approach may be especially helpful for patients undergoing their first biopsy or those being monitored with active surveillance. Our results suggest that using both methods together can offer the most complete and reliable diagnosis for prostate cancer.

## 1. Introduction

Prostate cancer (PCa) remains the second-most frequently diagnosed malignancy and the fifth leading cause of cancer-related mortality in men worldwide [1]. Its clinical course is highly variable, ranging from indolent tumors suitable for active surveillance to aggressive disease requiring immediate treatment. This heterogeneity highlights the need to accurately distinguish clinically significant prostate cancer (csPCa), usually defined as ISUP-grade group ≥ 2, from low-risk disease that may not require immediate intervention. Overdiagnosis of insignificant cancer can lead to overtreatment, exposing patients to unnecessary risks and side effects [2,3].

Multiparametric magnetic resonance imaging (mpMRI) has become a cornerstone in the diagnostic workup of PCa, enabling non-invasive detection, localization, and risk stratification of prostate lesions. mpMRI demonstrates high sensitivity for detecting csPCa, especially in lesions exceeding 10 mm in diameter, and has contributed to a paradigm shift in biopsy guidance strategies [4]. When suspicion arises due to elevated prostate-specific antigen (PSA) levels or an abnormal digital rectal examination (DRE), mpMRI is now a widely accepted next diagnostic step [5,6].

MRI-targeted biopsy (MRI-TB)—whether performed via cognitive guidance, MRI–ultrasound fusion software, or direct in-bore techniques—has been shown to improve the detection of csPCa and reduce the diagnosis of ISUP grade 1 cancers, thereby mitigating the risk of overtreatment when compared to conventional systematic biopsy (SB) [7,8,9,10]. Accordingly, both the American Urological Association (AUA) and European Association of Urology (EAU) recommend performing MRI-TB in patients with suspicious lesions on MRI, both in biopsy-naïve patients and in those with previous negative biopsies [5,6].

Despite the advantages of MRI-TB, the role of systematic biopsy (SB) remains contentious. While SB may increase the detection of insignificant cancers and lead to overtreatment [11,12], it may also uncover csPCa that is missed by targeted approaches [7]. This has led to ongoing debate about the added value of combining MRI-TB with SB, especially in the context of imaging-guided precision medicine.

To address this controversy, we conducted a retrospective study at our institution where transperineal MRI–US fusion-guided biopsies are routinely performed combined with systematic sampling to ensure maximal detection of csPCa. In this study, we evaluated the diagnostic accuracy and histological outcomes of targeted biopsy, systematic biopsy, and their combination in detecting PCa and csPCa. We also examined the correlation between PI-RADS scores and biopsy results, providing insight into the real-world accuracy of mpMRI-guided diagnostics. Our findings aim to clarify the relative contributions of targeted and systematic biopsy approaches and inform clinical decision making in prostate cancer diagnostics.

## 2. Materials and Methods

We retrospectively analyzed 356 patients who underwent transperineal MRI-TB and SB at our hospital between 2020 and 2023. Patients with an elevated PSA level or suspicious DRE and having at least one lesion Prostate Imaging-Reporting and Data System (PI-RADS) score of ≥3 were included in the study. All mpMRI examinations were interpreted by certified radiologists, specializing in genitourinary radiology. PI-RADS v2.1 scores were assigned prospectively as part of the clinical routine. All mpMRI examinations were performed on 3 Tesla scanners (Siemens Healthineers or GE Healthcare) using a standardized protocol in accordance with PI-RADS v2.1 recommendations. Sequences included high-resolution T2-weighted imaging (T2WI) in sagittal, axial, and coronal planes; diffusion-weighted imaging (DWI) with b-values of 0, 100, and 1000 and calculated 1500 s/mm^2^; and dynamic contrast-enhanced (DCE) imaging with 12 temporal phases (1 every 15 s) after two baseline acquisitions. The acquisition parameters are summarized in Supplementary Table S1. All scans used identical parameters across patients to ensure reproducibility. Additionally, patients with previous negative biopsy or in active surveillance were also included. All lesions detected by MRI were biopsied via the transperineal approach using the KOELIS MRI-US fusion platform (Koelis Trinity, Meyland, France). The systematic biopsies were performed with the transperineal approach in the same session, without avoiding the target lesions. The systematic biopsy protocol was uniform across patients but adjusted for prostate volume. For prostates ≤ 80 g, 16 cores were obtained—4 from the anterior region and 4 from the posterior region of each lobe. For prostates > 80 g and longer than 3 cm in longitudinal dimension, an additional 4 basal cores per lobe were taken, resulting in 24 total cores. This sampling strategy ensured adequate representation of both anterior and posterior regions, as well as basal areas in larger glands. The interval between mpMRI and biopsy ranged from 2 to 5 months, with no intervening therapy during this period.

The following baseline and clinical characteristics were noted for all eligible: age, PSA, prostate volume (PV), PSA density (PSAD), number of lesions, MRI location of lesions, MRI size of lesions, and PI-RADS score of the lesions. The following histopathological data were collected: Gleason score and ISUP grade total of each lesion and separated by MRI-TB and SB.

For this study, csPCa was defined by the presence of a Gleason score of 3 + 4 (ISUP 2) or higher. The ISUP grades were assigned according to the 2014 ISUP Consensus Conference, as follows: ISUP1 = Gleason score 6, ISUP 2 = 3 + 4, ISUP 3 = 4 + 3, ISUP 4 = Gleason score 8, and ISUP 5 = Gleason scores 9–10 [13]. The MRI-TB was performed using the Koelis system under sedation and outpatient regimen.

Statistical analyses were performed to evaluate the diagnostic performance of MRI–US fusion-TB, SB, and the combination of both techniques for prostate cancer detection. Descriptive statistics were used to summarize the patient demographics and lesion characteristics, including the median and interquartile range (IQR) for continuous variables and frequencies with percentages for categorical variables. The Wilcoxon rank sum test, Pearson’s chi-squared test, and Fisher’s exact test were used to check for statistical significance as appropriate.

For group comparisons, Pearson’s chi-squared test was used to assess differences in detection rates of prostate cancer, PCa and csPCa, between TB and SB. The sensitivity, specificity, and overall accuracy of each biopsy approach were calculated using the combined result of the TB and SB as the reference standard. Receiver operating characteristic (ROC) curve analysis was also performed to evaluate and compare the discriminatory performance of TB and SB for cancer detection.

The ISUP grade concordance between targeted and systematic biopsies was analyzed to determine the proportion of lesions with identical grades, upgrades (ISUP higher in TB), or downgrades (ISUP higher in SB). These are reported as absolute counts and percentages.

Subgroup analyses were performed by stratifying lesions according to the following clinical indications: patients under active surveillance (AS) and patients undergoing diagnostic biopsy. All statistical tests were two-tailed, and a *p*-value of <0.05 was considered statistically significant.

All analyses were performed using R statistical software, version 4.5.0 (R Foundation for Statistical Computing, Vienna, Austria, https://www.R-project.org, accessed on 13 May 2025).

## 3. Results

A total of 356 patients underwent MRI–US fusion-targeted biopsy (TB) and systematic biopsy (SB), with a combined total of 452 lesions analyzed. The baseline clinical and imaging characteristics of the 452 lesions are summarized in Table 1.

### 3.1. Study Cohort Characteristics

The median age of the overall population was 68 years (IQR: 62–74). PSA density had a median value of 0.11 ng/mL/cm^3^ (IQR: 0.07–0.17), and serum PSA levels had a median of 6.1 ng/mL (IQR: 4.6–8.4). Age, PSA, and PSA density did not differ significantly between patients under active surveillance and those undergoing initial biopsy (all *p* > 0.05).

In the overall cohort, 290 lesions (64%) were from patients with only one suspicious lesion, while 162 lesions (36%) were from patients with two lesions on MRI.

Regarding prior biopsy status, 255 lesions (56%) came from patients without previous biopsy, and 108 lesions (24%) were from patients with a prior negative biopsy. Notably, 89 lesions (20%) were from patients under active surveillance, all of whom had previously confirmed PCa.

The following are reported in terms of lesion risk stratification by PI-RADS score:

- PI-RADS 3: 88 lesions (20%);
- PI-RADS 4: 309 lesions (68%);
- PI-RADS 5: 55 lesions (12%).

There were no significant differences in PI-RADS distribution between patients under surveillance and the rest of the cohort (*p* = 0.7).

Lesions were also categorized by simplified anatomical location, as follows:

- Peripheral zone: 337 lesions (75%);
- Transitional/central zone: 115 lesions (25%).

No statistically significant differences were observed between groups in terms of the simplified location of the lesion (*p* = 0.5).

Of the 452 lesions analyzed, 323 (71%) were diagnosed as prostate cancer (PCa). Among these, 223 lesions (49%) were classified as clinically significant PCa (csPCa), defined as an ISUP grade ≥ 2, while 100 lesions (22%) were non-clinically significant (ISUP 1). The remaining 129 lesions (29%) were benign.

When stratified by clinical context, lesions from patients under active surveillance had a higher overall cancer detection rate, with 82 of 89 lesions (92%) showing PCa compared to 66% (241/363) in patients undergoing initial or repeat diagnostic biopsy (*p* < 0.001). However, the proportion of csPCa among positive cases was similar between the groups, as follows: 65% (53/82) in the AS group and 71% (170/241) in the diagnostic group (*p* = 0.3). These findings suggest that despite a prior cancer diagnosis, a substantial proportion of active surveillance patients harbored csPCa at re-biopsy, supporting the continued role of combined biopsy techniques in this subgroup.

### 3.2. Cancer Detection Rates of Targeted and Systematic Biopsies

Targeted biopsy detected PCa in 286 of 452 lesions (63%), while systematic biopsy identified PCa in 260 of 452 lesions (58%). Although TB had a slightly higher detection rate, the difference was not statistically significant (*p* = 0.077).

For csPCa, TB detected 191 lesions (42%), significantly more than the 151 lesions (33%) identified by SB (*p* = 0.023), indicating greater efficacy of the targeted approach in detecting clinically relevant disease (Table 2).

#### 3.2.1. Diagnostic Performance

The diagnostic performance of each biopsy technique for detecting csPCa was evaluated using the combined results of both the targeted and systematic biopsies as the reference standard. As shown in Table 3, targeted biopsy demonstrated a sensitivity of 93.7%, a specificity of 66.4%, and an overall accuracy of 79.9%. In comparison, systematic biopsy yielded a sensitivity of 85.7%, a specificity of 69.9%, and an accuracy of 77.6%. These results highlight the higher sensitivity and overall diagnostic accuracy of targeted biopsy, while systematic biopsy provided a marginally higher specificity.

**Figure 1:**
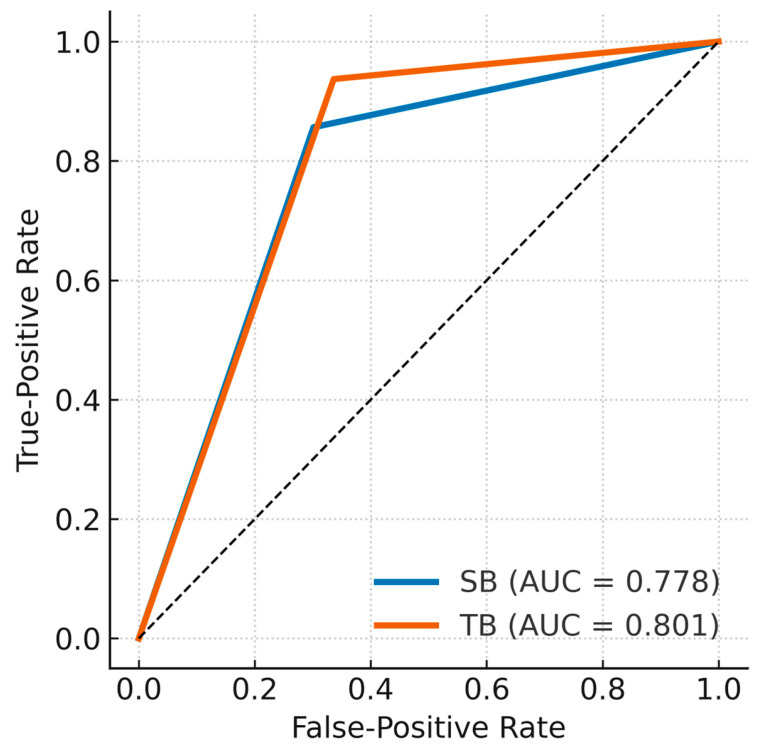
ROC curves for the detection of prostate cancer at the lesion level using SB and TB, with the combined approach serving as the reference standard. Areas under the curve (AUCs) were 0.778 for SB and 0.801 for TB. The combined method is not displayed because it was considered the gold standard.

The ROC analysis for the detection of prostate cancer showed an area under the curve (AUC) of 0.778 for SB and 0.801 for TB, with TB demonstrating higher discriminatory ability for cancer detection (Figure 1).

#### 3.2.2. Additional Value of Systematic Biopsy

While MRI–US fusion-targeted biopsy demonstrated superior sensitivity and clinically significant cancer detection overall, systematic biopsy still provided relevant diagnostic benefit. Among all lesions, 37 (8.2%) were diagnosed exclusively by systematic biopsy and would have been missed had only targeted sampling been performed.

**Figure 2:**
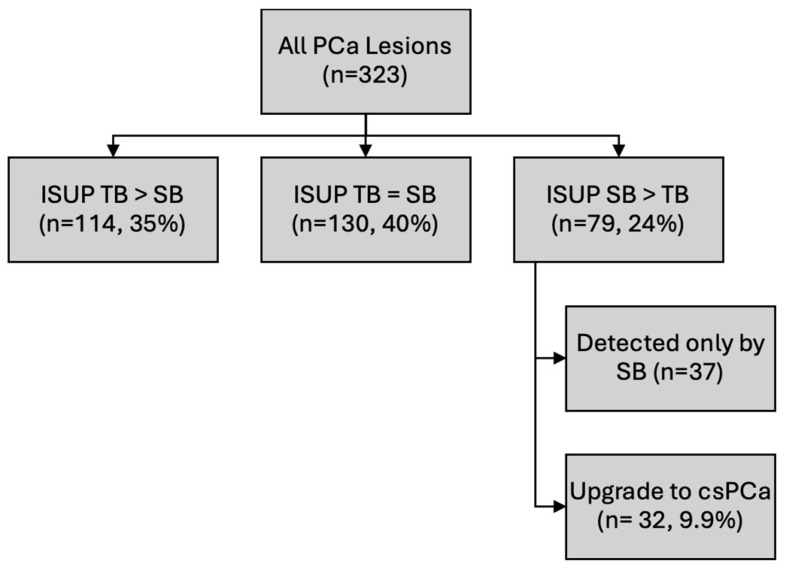
ISUP grade comparison and the additional value of systematic biopsy. Comparison of the ISUP-grade group results between MRI–US fusion-TB and SB for all PCa lesions (*n* = 323). Lesions were categorized as ISUP TB > SB (*n* = 114, 35%) when TB assigned a higher ISUP grade than SB, ISUP TB = SB (*n* = 130, 40%) when TB and SB yielded the same ISUP grade, or ISUP SB > TB (*n* = 79, 24%) when SB assigned a higher ISUP grade than TB. Within the latter group, 37 lesions were detected exclusively by SB, and 32 lesions (9.9%) were upgraded to csPCa (ISUP ≥ 2) based on SB findings.

Moreover, the ISUP grade comparison between the biopsy methods revealed that for 79 lesions (24%), the ISUP grade was higher on systematic biopsy than on targeted biopsy. Importantly, 32 lesions (9.9%) were classified as csPCa, indicating the role of systematic biopsy in detecting not only additional cancers but also tumors of higher prognostic relevance that may alter clinical management (Figure 2).

### 3.3. Correlation Between PI-RADS Score and Detection of Clinically Significant Cancer

**Figure 3:**
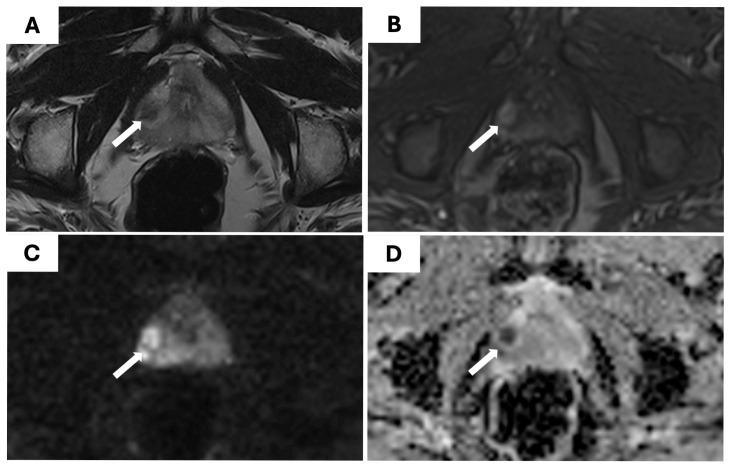
Representative case of a PI-RADS 4 lesion (white arrows): (**A**) axial T2-weighted MRI showing a hypointense lesion in the right peripheral zone; (**B**) dynamic contrast-enhanced image showing early enhancement; (**C**) diffusion-weighted imaging (DWI) b1500 revealing high signal intensity in the same region; (**D**) apparent diffusion coefficient (ADC) map showing marked hypointensity, consistent with restricted diffusion. The lesion was classified as PI-RADS 4 and confirmed as ISUP grade 2 adenocarcinoma on targeted and systematic biopsies.

A statistically significant correlation was found between the PI-RADS score on mpMRI and the likelihood of detecting clinically significant prostate cancer (csPCa) (*p* < 0.001). Among the 88 lesions scored as PI-RADS 3, csPCa was identified in 23% (20/88). This rate increased markedly for PI-RADS 4 lesions, with 53% (163/309) harboring csPCa, and was the highest among the PI-RADS 5 lesions, where 73% (40/55) were clinically significant. These findings confirm the strong predictive value of the PI-RADS classification system for stratifying cancer risk and guiding biopsy decisions. The diagnostic accuracy of mpMRI is shown in Table 4. An example of a PI-RADS 4 lesion detected on mpMRI and confirmed as csPCa on biopsy is shown in Figure 3.

## 4. Discussion

The integration of mpMRI into the diagnostic pathway for PCa has significantly improved the detection of csPCa while reducing the overdiagnosis of indolent disease [4,7,10,14]. Our findings support this paradigm shift, demonstrating that MRI–US fusion-guided TB outperforms SB in detecting csPCa, with a sensitivity of 93.7% versus 85.7%, as well as an overall accuracy of 79.9% versus 77.6%, respectively. These findings are consistent with prior evidence, including the PRECISION randomized trial, which showed that an MRI-directed pathway with targeted biopsy increases csPCa detection and reduces the diagnosis of ISUP grade 1 cancers compared with standard biopsy [10,15].

Despite the superior performance of TB, SB continues to play a complementary role. In our cohort, 8.2% of csPCa cases were detected exclusively by SB, and 9.9% of lesions were upgraded in ISUP grade by SB compared to TB. These findings are consistent with a growing body of evidence highlighting the risk of omitting systematic sampling. A large prospective study found that systematic biopsies provided an incremental csPCA detection of 10.4% in biopsy-naïve patients [16]. A recent review summarized that the added value of systematic biopsy ranges from 5 to 16% across various studies [17]. This incremental value is crucial, as a meta-analysis calculated that an MRI-guided biopsy-only approach would miss 19% of all prostate cancers and, more importantly, 10% of clinically significant cases [18]. Our results are in line with previous studies that emphasize that SB can identify significant lesions missed by TB, particularly multifocal or anterior lesions [9,12,17,19,20,21,22,23,24]. The European Association of Urology (EAU) and American Urological Association (AUA) guidelines continue to recommend combined biopsy approaches in certain clinical contexts [5,6].

The correlation between PI-RADS scores and csPCa detection observed in our study further validates the predictive value of mpMRI. The detection rate of csPCa increased from 23% in PI-RADS 3 lesions to 73% in PI-RADS 5 lesions, mirroring trends reported in earlier prospective studies [8,25]. This supports the use of mpMRI not only for lesion localization but also for risk stratification and biopsy decision making [26,27]. This interpretation is consistent with the PROMIS study, which—using pre-biopsy mpMRI—demonstrated high sensitivity and negative predictive value for csPCa and supported the role of mpMRI to safely avoid unnecessary biopsies when imaging is negative [15]. Nonetheless, csPCa was still present in a notable proportion of PI-RADS 3 lesions (23%), highlighting that mpMRI is not infallible and that systematic sampling retains diagnostic value in patients with low-to-intermediate imaging scores. This is particularly true when clinical risk factors are elevated; multivariable analyses have consistently shown that factors such as PSA density and age are significantly associated with csPCa detection on biopsy [11,16,26,27].

The clinical implications of these findings extend beyond initial diagnosis, particularly for treatment planning and active surveillance. Systematic biopsy plays a crucial role in identifying multifocal disease that may not be visible on MRI, and missing these secondary lesions can lead to treatment failure [17]. Our inclusion of patients under AS adds valuable insight into this subgroup. Notably, 65% of AS patients with positive biopsies harbored csPCa, underscoring the importance of continued monitoring and the potential role of mpMRI and combined biopsy in re-evaluation. These findings align with current EAU and AUA guidelines, which recommend mpMRI in AS protocols to guide re-biopsy decisions [5,6].

The transperineal approach used in our study offers additional advantages, including reduced infection risk and improved access to anterior lesions, as supported by the recent literature [28,29,30]. Moreover, the use of MRI–US fusion software enhances targeting precision, although inter-reader variability and operator experience remain challenges [31,32].

Beyond diagnostic performance, MRI-targeted strategies have also been associated with fewer biopsy cores and complications, contributing to more patient-centered and cost-effective care [14,19]. Future diagnostic algorithms may benefit from integrating mpMRI findings with clinical risk factors such as PSA density and lesion location to personalize the use of SB and reduce unnecessary sampling without compromising cancer detection [5,11,14].

A key methodological aspect of our work is that the primary analysis was performed at the lesion level rather than the patient level. Lesion-based analysis enables a detailed evaluation of how individual imaging features correlate with histopathologic outcomes. However, this approach has limitations. In lesion-based comparisons, TB is performed for each MRI-identified lesion, whereas SB is performed once per patient. In patients with multiple lesions, this asymmetry can lead to an overestimation of TB performance and, conversely, lower apparent sensitivity and accuracy for SB.

In addition, targeted biopsy was always performed before systematic biopsy, which may have introduced a subtle operator bias. While systematic cores were obtained independently, the awareness of lesion location following TB may have unintentionally influenced sampling during SB, potentially increasing its detection yield. However, this reflects a real-world workflow used in many institutions.

## 5. Conclusions

MRI–US fusion-guided targeted biopsy significantly improves the detection of clinically significant prostate cancer compared to systematic biopsy alone. However, systematic biopsy continues to provide complementary diagnostic value, identifying additional csPCa cases and upgrading tumor grades in a meaningful subset of patients. The combination of both techniques offers the most comprehensive diagnostic yield, particularly in biopsy-naïve patients and those under active surveillance.

The strong association between PI-RADS score and csPCa detection supports mpMRI as a reliable triage tool. Nevertheless, the residual risk of missed disease, variability in imaging interpretation, and limitations of targeted sampling highlight the importance of maintaining a combined diagnostic approach, especially in equivocal cases.

Incorporating mpMRI and MRI–US fusion-guided biopsy into routine clinical practice represents a paradigm shift toward precision diagnostics in prostate cancer. Future prospective studies should evaluate long-term oncologic outcomes associated with different biopsy strategies and help define which patient subgroups may safely forgo systematic sampling without compromising diagnostic accuracy.

## Figures and Tables

**Table 1:** Baseline clinical, imaging, and histologic characteristics of the lesions.

Characteristic	Value
Age, median (IQR)	68 (62–74)
PSA density, median (IQR)	0.11 (0.07–0.17)
PSA, median (IQR)	6.1 (4.6–8.4)
Clinical Context	
No previous biopsy	255 (56%)
Previous biopsy: negative	108 (24%)
Previous biopsy: active surveillance	89 (20%)
Number of lesions on MRI	
1	290 (64%)
>1	162 (36%)
PI-RADS	
3	88 (20%)
4	309 (68%)
5	55 (12%)
Location on MRI	
Peripheral zone	337 (75%)
Transitional/central zone	115 (25%)
PCa	
Total	323 (71%)
Detected by TB	286 (63%)
Detected by SB	260 (58%)
csPCa	
Total	223 (49%)
Detected by TB	191 (42%)
Detected by SB	151 (33%)

IQR: interquartile range; PSA: prostate-specific antigen; MRI: magnetic resonance imaging; PCa: prostate cancer; TB: targeted biopsy; SB: systematic biopsy; csPCa: clinically significant PCa.

**Table 2:** Detection rates of csPCa for targeted vs. systematic biopsy.

	No Cancer	PCa		*p*-Value ^1^
		Non-csPCa	csPCa	
Method				0.023
Systematic	192 (42%)	109 (24%)	151 (33%)	
Targeted	166 (37%)	95 (21%)	191 (42%)	
Total	358 (40%)	204 (23%)	342 (38%)	

^1^ Pearson’s chi-squared test; PCa: prostate cancer; csPCa: clinically significant PCa.

**Table 3:** Diagnostic accuracy of TB vs. SB ^1^.

Method	Specificity	Sensitivity	Accuracy
Systematic	0.6987	0.8565	0.7765
Targeted	0.6638	0.9372	0.7987

^1^ Sensitivity, specificity, and accuracy in detecting csPCa, using the combined results of both methods as the reference standard.

**Table 4:** Diagnostic accuracy of mpMRI (PI-RADS vs. histology).

Characteristic	3 (N = 88) ^1^	4 (N = 309) ^1^	5 (N = 55) ^1^	*p*-Value ^2^
No PCa	41	87	1	<0.001
Non-csPCa (ISUP 1)	27 (57%)	59 (27%)	14 (26%)	
csPCa (ISUP ≥ 2)	20 (43%)	163 (73%)	40 (74%)	

^1^ n (%). ^2^ Pearson’s chi-squared test.

## Data Availability

The data presented in this study are not publicly available due to privacy and ethical restrictions. Access to anonymized datasets may be provided by the corresponding author upon reasonable request and pending institutional approval.

## Supplementary Materials

The following supporting information can be downloaded at: https://www.mdpi.com/article/10.3390/cancers17172735/s1, Table S1: MRI acquisition parameters.

## Abbreviations

The following abbreviations are used in this manuscript:

PCa	Prostate Cancer
csPCa	Clinically Significant Prostate Cancer
ISUP	International Society of Urological Pathology
mpMRI	Multiparametric Magnetic Resonance Imaging
PSA	Prostate-Specific Antigen
DRE	Digital Rectal Examination
MRI	Magnetic Resonance Imaging
MRI-TB	Magnetic Resonance Imaging–Targeted Biopsy
SB	Systematic Biopsy
EAU	European Association of Urology
AUA	American Urological Association
MRI–US	Magnetic Resonance Imaging–Ultrasound
TB	Targeted Biopsy
PV	Prostate Volume
PSAD	PSA Density
PI-RADS	Prostate Imaging Reporting and Data System
IQR	Interquartile Range
AS	Active Surveillance
TRUS	Transrectal Ultrasound
