# Supplementary material for: Transperineal MRI-US Fusion-Guided Biopsy with Systematic Sampling for Prostate Cancer: Diagnostic Accuracy and Clinical Implications Across PI-RADS
Source: Cancers (Basel). 2025 Aug 22;17(17):2735. doi: 10.3390/cancers17172735 (PMC12427366; doi:10.3390/cancers17172735)
Supplement: Supplementary file 1 [file cancers-17-02735-s001.zip › cancers-3769468-supplementary.pdf]

Supplementary Table S1. MRI acquisition parameters.

Sequence	Plane	Slice Thickness (mm)	FOV (mm)	Contrast	Butylscopolamine	Notes
**T2**	Sagittal	3.0	180	No	Yes	—
**T2**	Axial	3.0	180	No	Yes	—
**T2**	Coronal	3.0	180	No	Yes	—
**T1 Pelvis**	Axial	4.0	400	No	Yes	—
**DWI b0/100/1000 + calc 1500**	Axial	4.0	260	No	Yes	—
**Dynamic (12 phases)**	Axial	3.6	260	Yes	Yes	18 phases: 1 every 15s, 2 baseline; total ≈3:30 min; remove extra phases if longer; automatic subtraction
